# Supplementary material for: A pilot study investigating the effects of voluntary exercise on capillary stalling and cerebral blood flow in the APP/PS1 mouse model of Alzheimer’s disease
Source: PLoS One. 2020 Aug 28;15(8):e0235691. doi: 10.1371/journal.pone.0235691 (PMC7455035; doi:10.1371/journal.pone.0235691)
Supplement: S1 Fig — Both running (RUN) and sedentary (SED) APP/PS1 mice exhibited robust exploration, as assayed by the time spent exploring the old and replaced or new objects, in the A object replacement test, and B novel object recognition test. Running and sedentary mice also had C the same number of arm entries, on average, in the Y-maze task, and D the same average total track length in the open field test. (DOCX) [file pone.0235691.s001.docx]

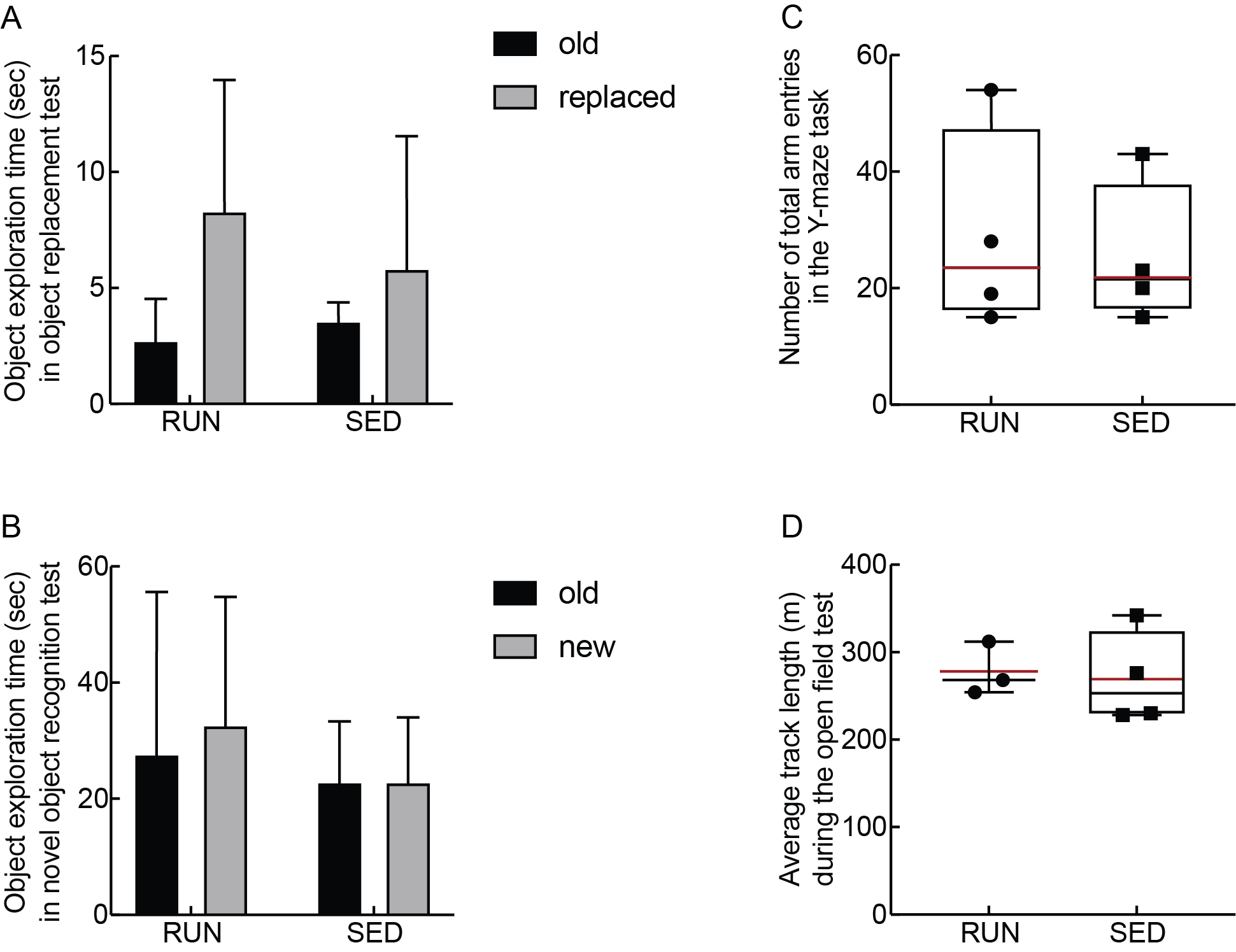


**S1 Fig. Running did not affect locomotor activity in behavioral assays.** Both running (RUN) and sedentary (SED) APP/PS1 mice exhibited robust exploration, as assayed by the time spent exploring the old and replaced or new objects, in the **A** object replacement test, and **B** novel object recognition test. Running and sedentary mice also had **C** the same number of arm entries, on average, in the Y-maze task, and **D** the same average total track length in the open field test.
