# Supplementary material for: A pilot study investigating the effects of voluntary exercise on capillary stalling and cerebral blood flow in the APP/PS1 mouse model of Alzheimer’s disease
Source: PLoS One. 2020 Aug 28;15(8):e0235691. doi: 10.1371/journal.pone.0235691 (PMC7455035; doi:10.1371/journal.pone.0235691)
Supplement: S2 Fig — Amyloid plaque density, represented as the number of plaques that were Methoxy-X04 positive; from the hippocampus (left) and cortex (right) of RUN and SED APP/PS1 mice. Animal numbers: RUN: n = 4; SED: n = 4. (DOCX) [file pone.0235691.s002.docx]

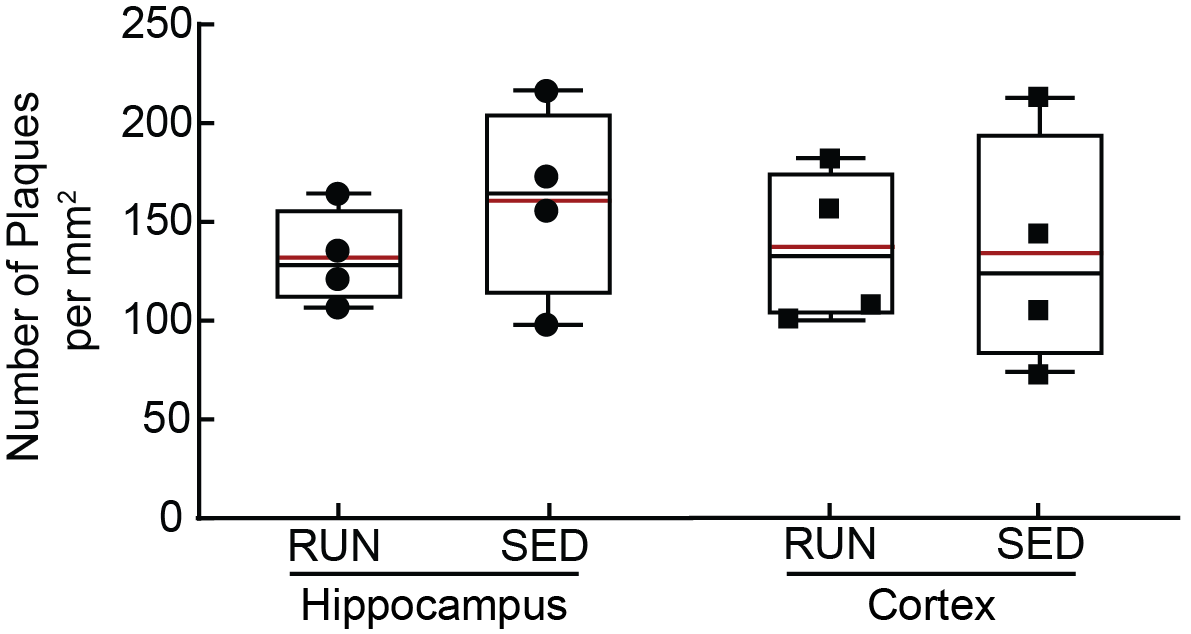


**S2 Fig. No difference was found in number of amyloid plaques.** Amyloid plaque density, represented as the number of plaques that were Methoxy-X04 positive; from the hippocampus (left) and cortex (right) of RUN and SED APP/PS1 mice. Animal numbers: RUN: *n* = 4; SED: *n* = 4.
