# Supplementary material for: A pilot study investigating the effects of voluntary exercise on capillary stalling and cerebral blood flow in the APP/PS1 mouse model of Alzheimer’s disease
Source: PLoS One. 2020 Aug 28;15(8):e0235691. doi: 10.1371/journal.pone.0235691 (PMC7455035; doi:10.1371/journal.pone.0235691)
Supplement: S3 Fig — Animal numbers: RUN: n = 4; SED: n = 4. (DOCX) [file pone.0235691.s003.docx]

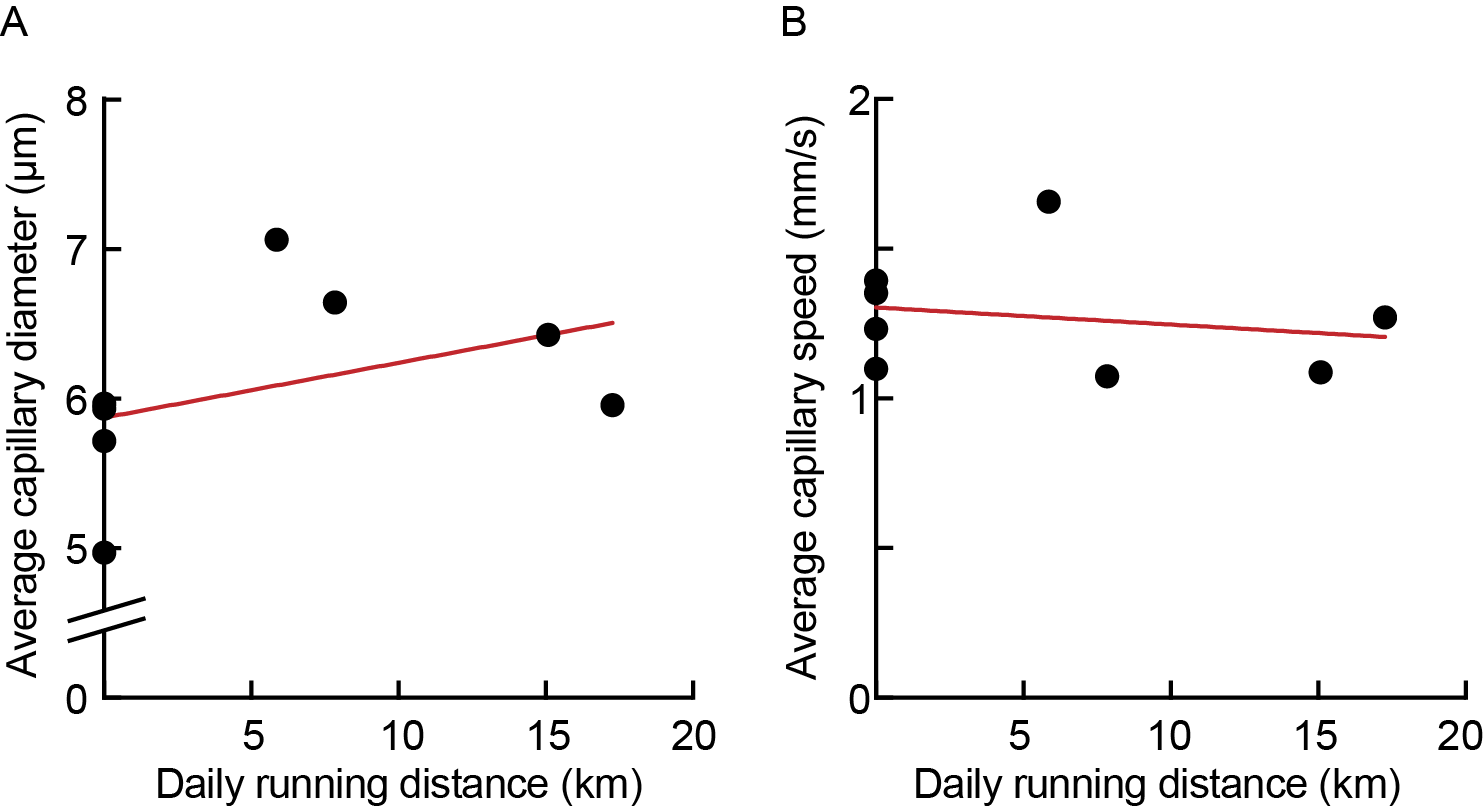


**S3 Fig. No correlation was found between the total running distance and the A average capillary diameter or B average capillary flow speed.** Animal numbers: RUN: *n* = 4; SED: *n* = 4.
