# Supplementary material for: A pilot study investigating the effects of voluntary exercise on capillary stalling and cerebral blood flow in the APP/PS1 mouse model of Alzheimer’s disease
Source: PLoS One. 2020 Aug 28;15(8):e0235691. doi: 10.1371/journal.pone.0235691 (PMC7455035; doi:10.1371/journal.pone.0235691)
Supplement: S4 Fig — A An example image of a line scan, showing the cells counted in a segment. B Average tube hematocrit in RUN and SED APP/PS1 mice. C Tube hematocrit as a function of capillary diameter. Animal numbers: RUN: n = 4; SED: n = 4. (DOCX) [file pone.0235691.s004.docx]

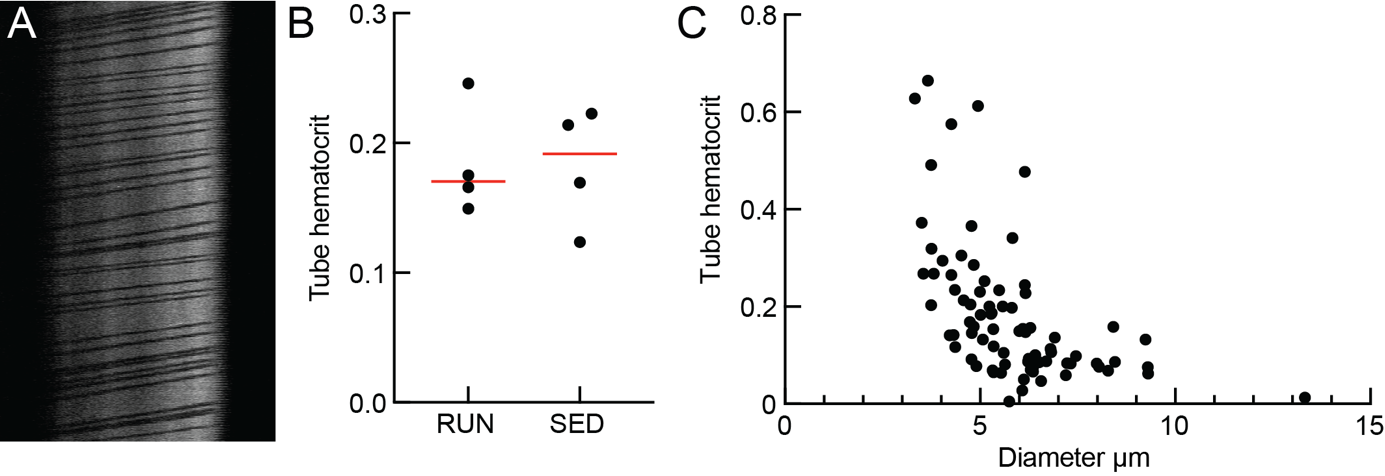


**S4 Fig. Capillary tube hematocrit from cortical microvascular network of running (RUN) and sedentary (SED) APP/PS1 mice.** **A** An example image of a line scan, showing the cells counted in a segment. **B** Average tube hematocrit in RUN and SED APP/PS1 mice. **C** Tube hematocrit as a function of capillary diameter. Animal numbers: RUN: n = 4; SED: n = 4.
