# Supplementary material for: A pilot study investigating the effects of voluntary exercise on capillary stalling and cerebral blood flow in the APP/PS1 mouse model of Alzheimer’s disease
Source: PLoS One. 2020 Aug 28;15(8):e0235691. doi: 10.1371/journal.pone.0235691 (PMC7455035; doi:10.1371/journal.pone.0235691)

**S1 Table. Characteristics of mice used in this study.** Running (n = 4) and sedentary (n = 4) mice of both sexes ranging from 10 to 13 months at the start of the study. Indicated is their average daily running distance in km.


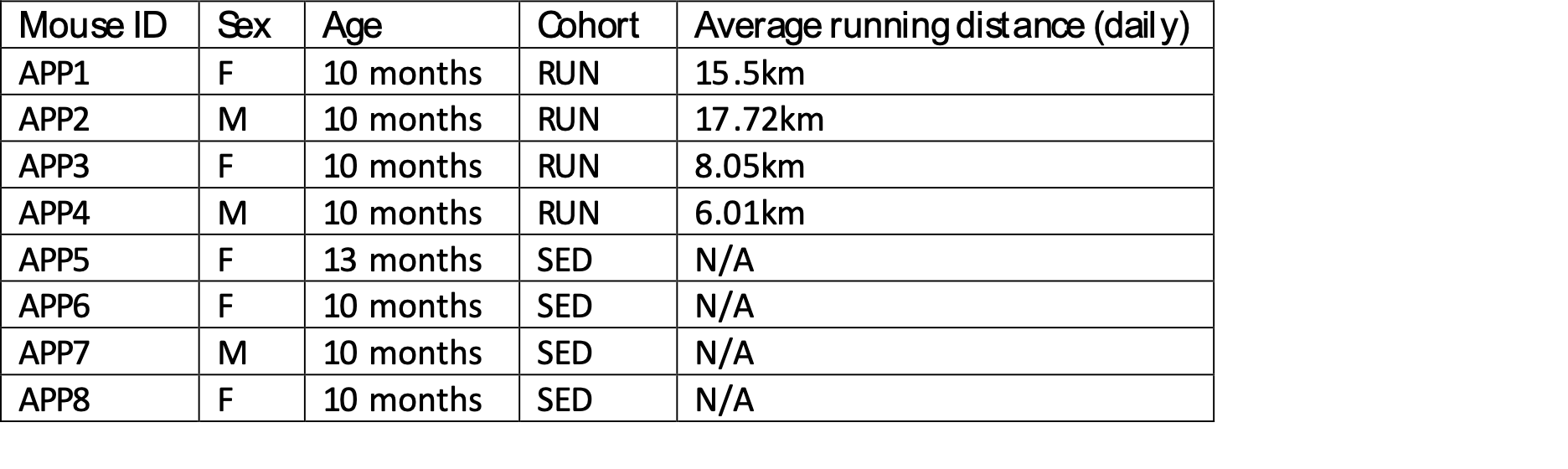

Supplement: S1 Table — Running (n = 4) and sedentary (n = 4) mice of both sexes ranging from 10 to 13 months at the start of the study. Indicated is their average daily running distance in km. (DOCX) [file pone.0235691.s005.docx]
